# Gelatin device for the delivery of growth factors involved in endochondral ossification

**DOI:** 10.1371/journal.pone.0175095

**Published:** 2017-04-05

**Authors:** Lucas A. J. Ahrens, Daniel Vonwil, Jon Christensen, V. Prasad Shastri

**Affiliations:** 1 Institute for Macromolecular Chemistry, Hermann Staudinger Haus, University of Freiburg, Freiburg, Germany; 2 BIOSS Centre for Biological Signalling Studies, University of Freiburg, Freiburg, Germany; National University of Ireland - Galway, IRELAND

## Abstract

Controlled release drug delivery systems are well established as oral and implantable dosage forms. However, the controlled release paradigm can also be used to present complex soluble signals responsible for cellular organization during development. Endochondral ossification (EO), the developmental process of bone formation from a cartilage matrix is controlled by several soluble signals with distinct functions that vary in structure, molecular weight and stability. This makes delivering them from a single vehicle rather challenging. Herein, a gelatin-based delivery system suitable for the delivery of small molecules as well as recombinant human (rh) proteins (rhWNT3A, rhFGF2, rhVEGF, rhBMP4) is reported. The release behavior and biological activity of the released molecules was validated using analytical and biological assays, including cell reporter systems. The simplicity of fabrication of the gelatin device should foster its adaptation by the diverse scientific community interested in interrogating developmental processes, *in vivo*.

## Introduction

Controlled release systems (CRS) have been in development since the middle of the 20^th^ century and have been successfully employed to deliver small molecules, peptides and proteins over extended periods of time ranging from a few days to months [1]. Notable examples are the delivery of leuprolide acetate from poly(lactic acid) to treat precocious puberty [2] and poly(lactic-*co*-glycolic acid) microspheres for the delivery of human growth hormone which is used to treat dwarfism in humans [3]. CRS have also been developed for the delivery of chemotherapeutic agents, for example the carmustine-releasing GLIADEL^®^ Wafer for the treatment of malignant glioma [4,5].

However, CRS are equally important as an investigative tool for the recapitulation of biological processes which are regulated through controlled spatial and temporal expression of a plethora of soluble molecules in specific patterns. CRS allow for the localized and controlled presentation of molecules to invoke these specific processes. In this regard, several systems have been developed for the delivery of proteins to induce angiogenesis [6,7] and bone formation [8,9]. More recently, CRS have been used to investigate the role of signaling gradients at play during the maturation of an embryo [10]. One of the processes during embryogenesis is endochondral ossification (EO), in which long bones are formed from aggregates of mesenchymal stem cells (MSC) through a series of biologically controlled events including an intermediate cartilage template [11]. This transformation is under control of many biological signals such as fibroblast growth factors (FGFs), Wingless-type MMTV integration site family members (WNTs), bone morphogenetic proteins (BMPs), vascular endothelial growth factors (VEGFs) and Indian hedgehog (IHH) which are temporally and spatially varied [12,13]. To date, a lot of effort has gone into understanding EO as a paradigm for developmental engineering [14]. The effort in this space has mainly focused on the delivery of single soluble signals, for example from microbeads [15,16] to perturb the endogenous signaling environment. But in order to exploit EO as a developmental engineering paradigm for the *de novo* engineering of either cartilage or bone, the precise interplay between these soluble signals has to be understood. Therefore, a system to deliver these soluble signals in a controlled fashion is crucial first step.

Historically, many drug delivery systems are based on synthetic polymers such as polydimethylsiloxane [17] or poly(ethylene-vinyl acetate) [18] because they offer an excellent possibility for the long term delivery of molecules. But in the context of delivering complex molecules in a sensitive environment such as tissue, a system that is capable of encapsulating a plurality of signals and is able to release all these signals independently while being compatible with the delivery space is essential. These prerequisites can also be met by polymers such as gelatin which has a history of use *in vivo* [19].

Gelatin type A and type B have a track record as an eclectic matrix material in drug delivery applications [20,21]. It offers several advantages as it can be sourced in high purity, is biocompatible, can be formulated in water and is easily processed. Therefore, gelatin-based delivery systems can be translated from the bench (*in vitro* tissue culture) to the clinic. Also, it is compatible with many soluble signals. A notable example of a commercialized gelatin-based CRS is PerioChip^™^, a system for the treatment of periodontitis through delivery of chlorhexidine to the periodontal pocket. In the field of bone tissue engineering, signaling compounds such as FGF2 [22–24], BMP4 [25,26] and VEGF [27,28] have been formulated and delivered from gelatin discs [23], fibers [29] or microparticles [28,30]. The necessary structural integrity at physiological conditions is typically obtained through crosslinking with glutaraldehyde (GA), either in solution [22] or by exposing the discs to GA vapor [31], although other compounds like genipin [32–34] or transglutaminase [35] are used as well.

Notwithstanding the long history of gelatin in drug delivery applications, there is no example to date demonstrating the development and validation of a comprehensive, easy-to-use gelatin-based system specifically aimed at delivering a library of EO-associated soluble signals. In this study, the controlled release of several soluble signals associated with EO, namely recombinant human (rh) FGF2, rhBMP4, rhVEGF, rhWNT3A, purmorphamine and a synthetic WNT agonist, from a GA-crosslinked gelatin type B matrix is presented (Table 1). Release durations ranging from few days to few weeks with the retention of biological activity in cellular and chick chorioallantoic membrane (CAM) assays is demonstrated.

**Table 1:** Properties and function of soluble signals involved in endochondral ossification.

Soluble Signal	Molecular weight (Da)	Biological function	Typical concentration in cell culture	Calculated isoelectric point (IP)
WNT agonist	350	Synthetic agonist on the Wnt pathway [36]	0.25 μg/ml	
Purmorphamine	521	Synthetic agonist on the Hh pathway [37–39]	0.5 μg/ml	
rhWNT3A	37,400	Acting on the Wnt pathway [40,41]	5–200 ng/ml	8.46
rhFGF2	16,400	Inhibits chondrocyte proliferation and facilitates hypertrophy during EO [42]	1–10 ng/ml	9.59
rhVEGF	19,000	Permits vascularization of cartilage [43]	1–10 ng/ml	9.01
rhBMP4	13,100	Acting on the TGF-ß pathway and acts during bone formation [44,45]	1–50 ng/ml	7.60

## Materials and methods

Water was deionized with a Milli-Q Reference water purification system manufactured by Millipore Corporation. Gelatin from bovine skin (type B, suitable for cell culture, gel strength of 225 g Bloom, #G9391), L-ascorbic acid 2-phosphate sesquimagnesium salt hydrate (AA), fluorometric aldehyde assay kit (MAK141) and dimethyl sulfoxide (DMSO) were purchased from Sigma-Aldrich. Glutaraldehyde solution (25% for electron microscopy) was procured from AppliChem. WNT agonist (#681665) was purchased from Calbiochem. Purmorphamine (#10009634) was procured from Cayman Chemical. Recombinant human FGF2 (#233-FB), rhBMP4 (314-BP), rhVEGF 165 (#293-VE), rhWNT3A (#5036-WN), the DuoSet enzyme-linked immunosorbent assay (ELISA) kits for human FGF2 (#DY233), human BMP4 (#DY314) and human VEGF 165 (#DY293B), as well as the human phospho-Smad1 (S463/S465)/Smad5 (S463/S465) cell-based ELISA (#KCB7660) were obtained from R&D Systems. The ELISA kit for rhWNT3A (#E83155Hu) was obtained from USCN Life Science Inc. Phosphate-buffered saline (PBS, #14190–094), Dulbecco’s modified Eagle medium (DMEM, #31966–021), minimum essential medium α (α-MEM, #22571–020), Rosewell Park Memorial Institute medium (RPMI, #61870–010), penicillin/streptomycin (PS, #15140–122), and fetal bovine serum (FBS, #10270) were supplied from Gibco. 4-(2-hydroxyethyl)-1-piperazineethanesulfonic acid (HEPES) buffer was purchased from PAN-Biotech. Bovine serum albumin (BSA, #126593) was obtained from Merck. Human serum albumin (HSA) was purchased from Baxter. The WNT sensitive lentiviral vector 7TGC was a gift from Roel Nusse (Addgene plasmid # 24304) [46]. Fluorescence microscopy was performed on a Zeiss Observer.Z1 equipped with a 20 x long distance objective. Further spectroscopic measurements were carried out on a BioTek Synergy HT microplate reader. Scanning electron microscopy (SEM) was performed on a FEI Quanta 250 FEG microscope using an accelerating voltage of 20 kV in high vacuum. The micrographs were then analyzed with xT Microscope Control (4.1.4). High-performance liquid chromatography (HPLC) was performed on a Dionex UltiMate 3000 device using a C18 column and UV-Vis detection.

### Cell culture

All cells were cultured at 37°C with 5% CO_2_. Human bone marrow mesenchymal stem cells (hMSCs) as well as hMSC-based WNT reporter cells were cultured in α-MEM with 10% FBS (v/v), 1% PS (v/v), AA (0.1 mmol/l) and rhFGF2 (5 ng/ml) when seeded in 2D. For pellet culture and other 3D applications, hMSC were cultured in DMEM with 2% FBS (v/v), 1% PS (v/v), HEPES (10 mmol/l), AA (0.1 mmol/l) and rhFGF2 (5 ng/ml). Human embryonic kidney (HEK) cells HEK 293T and HEK-based WNT reporter cells were cultured in DMEM with 10% FBS (v/v) and 1% PS (v/v).

### Preparation and characterization of delivery discs

**Fig 1:**
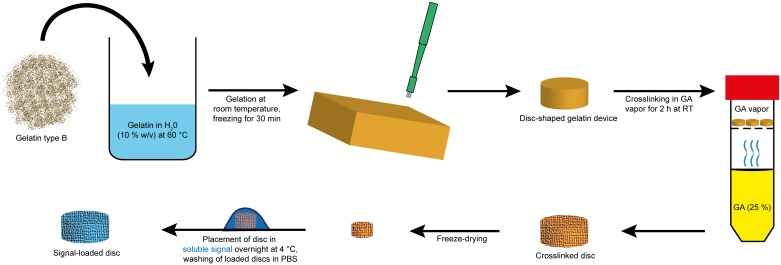
Schematic of the process steps in the preparation of the delivery disc. Disc-shaped devices were prepared by crosslinking gelatin in GA vapor followed by freeze-drying. Loading of the soluble signal into the disc was achieved by soaking overnight in solution of the soluble signal.

**Fig 2:**
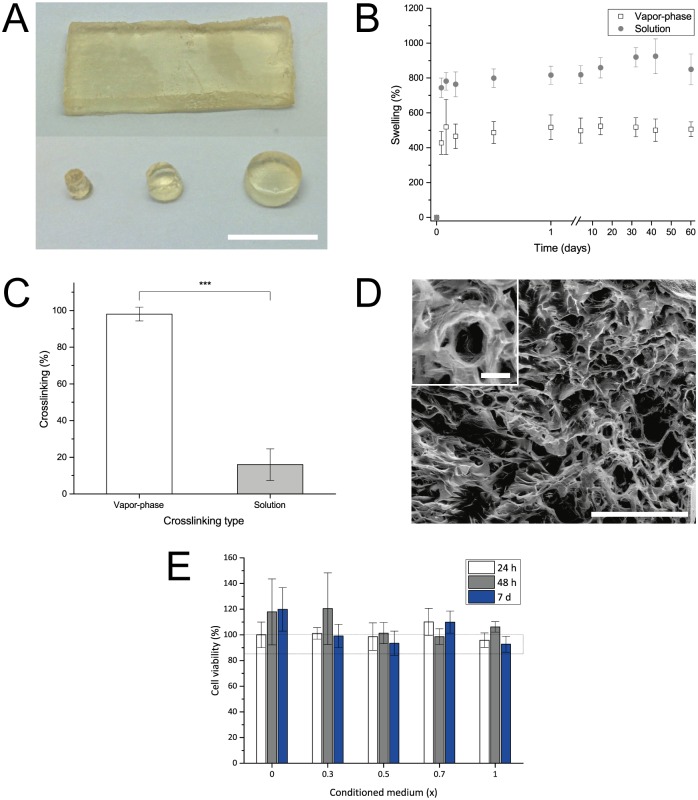
Characterization of gelatin delivery discs. **A** Different shapes and sizes of discs were obtainable. Scale bar equals 10 mm. **B** Discs crosslinked in vapor-phase (white) and solution (gray) were stable over weeks upon incubation in PBS. Both types showed a high degree of swelling in the first hours of incubation due to rehydration. Data is presented as mean ± SD, n = 6. **C** Degree of crosslinking determined for gelatin crosslinked in the vapor-phase (white) and solution (gray). Data is presented as mean ± SD, n = 6. **D** SEM micrograph of a vapor-phase crosslinked disc’s interior, revealing a porous structure. Scale bar equals 500 μm. Scale bar in inset (3x) equals 50 μm. **E** hMSCs cultured in delivery disc-conditioned medium remained viable over 7 days, as assessed using a MTT assay. Data is presented as mean ± SD, n = 3.

The process of preparing the gelatin delivery discs is shown in Fig 1. Gelatin powder (200 mg, 10% (w/v)) was suspended in Milli-Q water (2.0 ml) and heated to 60°C. Once completely dissolved, the solution was cast into a plastic dish for gelation at room temperature (RT) and then frozen for 30 min. Disc-shaped delivery devices were punched out of the frozen gelatin slab with a biopsy punch. The dimensions of the discs were controlled by the gel thickness and the diameter of the biopsy punch (Fig 2A). The discs were then crosslinked at RT for 2 h in glutaraldehyde vapor in a closed container and freeze-dried overnight thereafter. The discs were stored at -20°C until further use.

The resistance of discs towards temperature and water was tested by placing freeze-dried discs with known weight in PBS at 37°C. At predefined time points, the discs were blotted dry, weighed again, and placed in PBS. The swelling of each disc was then calculated as a ratio of the actual to the initial weight.

The degree of gelatin crosslinking was determined by quantifying the amount of free amino groups in gelatin before and after crosslinking using 2,4,6-Trinitrobenzenesulfonic acid (TNBS) and following a published protocol [47]. The degree of crosslinking was normalized to untreated gelatin.

To determine free glutaraldehyde in crosslinked gelatin discs crosslinked and un-crosslinked (control) discs were incubated in 500 μl of water at 37°C for 1 h and the supernatant was collected and quantified using a fluorometric assay as per the protocol of the kit supplier. Concentration of glutaraldehyde then was determined using a calibration standard of freshly prepared glutaraldehyde of known concentration.

Cytotoxicity of the prepared gelatin discs was assessed using a 3-(4,5-dimethylthiazol-2-yl)-2,5-diphenyltetrazolium bromide (MTT) assay. In short, gelatin discs were placed in 2.5 ml of DMEM (supplemented with 10% serum) each for 5 days at 37°C in order to leach out any toxic components hMSCs (2000 cells/well in a 96 well plate) were cultured in presence of this extract (1.0 x) and a serial dilution of this extract with α-MEM (0.7 x, 0.5 x, 0.3 x and 0.0 x) for 1, 2 and 7 days. The cell metabolic activity was then determined following a standard MTT protocol.

### Quantification of signal release

The delivery discs were UV-sterilized for 2 h. A typical delivery disc (2 × 2 mm) with a volume of 6.28 μl was placed in 5 μl of soluble signal solution with the desired concentration overnight at 4°C for complete absorption. While the proteins were reconstituted in PBS containing 0.1% BSA prior to use, WNT agonist and purmorphamine were dissolved in DMSO. In case of rhFGF2, a formulation with heparin and BSA (1:100:1000 w/w with respect to rhFGF2) was prepared before loading which was adapted from procedures described elsewhere [48,49]. The thermal stability of rhFGF2 and rhBMP4 before and after formulation was assessed in DMEM by placing solutions of both conditions at 37°C. The change in protein concentration was determined daily using an ELISA kit.

Loaded discs were washed in PBS (2 × 1 ml) and then placed separately in 500 μl of DMEM with 10% FBS (v/v) and 1% PS (v/v) at 37°C with 5% CO_2_. At predefined time points, the supernatant was removed, stored at -150°C and replaced with fresh, preheated medium. The supernatants of WNT agonist and purmorphamine release experiments were analyzed using HPLC. The supernatants of the rhFGF2, rhBMP4, rhVEGF and rhWNT3A release experiments were analyzed using ELISA kits according to the manufacturer’s manual. Recombinant human WNT3A supernatants were additionally analyzed using a WNT reporter cell assay that was developed in-house. 7TGC lentiviral particles were produced in HEK 293T cells and used for transduction as previously described [50]. Transduced cells express mCherry constitutively and eGFP on a WNT promoter upon intracellular activation of the Wnt pathway. These HEK reporter cells were seeded into a 96 well plate with a density of 30,000 cells/cm^2^. Two days after seeding, the medium was replaced with the collected supernatant as well as a standard ranging from 0 to 2000 ng/ml rhWNT3A, all in duplicates. The well plate was incubated at 37°C for 24 h and mCherry and eGFP fluorescence was then quantified by microscopy (S2A Fig). During imaging, more than 50% of the well surface was automatically scanned using the MosaiX plugin of the AxioVision software, resulting in a stitched image, which was converted into black and white. The fluorescence was then quantified through the amount of white pixels. In order to factor in differences in cell population, the eGFP signal was normalized to mCherry. The rhWNT3A concentration [rhWNT3A] in the unknown samples was calculated using a linear fit (Formula 1) for the averaged values of the standard curve (see Formula 2).

$$\left(\frac{eGFP_{Standard}}{mCherry_{Standard}}-\frac{eGFP_{Blank}}{mCherry_{Blank}}\right)=a+b\times {\left[rhWNT3A\right]}_{Standard}$$ (1)

$${\left[rhWNT3A\right]}_{Sample}=\frac{\left(\frac{eGFP_{Sample}}{mCherry_{Sample}}-\frac{eGFP_{Blank}}{mCherry_{Blank}}\right)-a}{b}$$ (2)

### Bioactivity of released signal

WNT3A: The bioactivity of rhWNT3A was determined with human WNT reporter MSCs transduced similar to the HEK-based WNT reporter cell line described above. These cells were cultured in well plates as well as in pellet culture and treated with rhWNT3A-loaded discs. The cellular response was analyzed 72 h after addition of the delivery discs.

FGF2 and VEGF: The bioactivity of rhFGF2 and rhVEGF was analyzed using the chick chorioallantoic membrane (CAM) assay, which allows following the blood vessel formation in fertilized eggs. Eggs were first bred three days *in* and then 7 days *ex ovo* at 37°C and an air humidity of 60%. Gelatin discs loaded either with 150 ng of rhFGF2 or rhVEGF per disc were then placed on the CAM for four days. Stereoscopic pictures were taken at days 0 and 4. For rhFGF2, the amount of blood vessels touching the delivery disc were counted and compared to control experiments with PBS-loaded discs. For VEGF, the branching of already present blood vessels was compared to control experiments with PBS-loaded discs.

BMP4: Bioactivity of rhBMP4 was ensured using a phospho-SMAD1/SMAD5 ELISA kit. Gelatin discs were loaded either with 0, 100 or 1000 ng/disc rhBMP4 and then incubated separately in 1 ml of α-MEM for 24 h at 37°C leading to solutions with rhBMP4 concentrations of roughly 0, 10 and 100 ng/ml. The conditioned medium was used to stimulate hMSCs for 30 min. Subsequent phosphorylation of SMAD1 and SMAD5, a cellular response downstream of the BMP pathway, was quantified following the instructions of the ELISA kit and compared to results obtained by direct addition of rhBMP4 to the culture medium.

### Data analysis

All values are reported as mean ± standard deviation and p-values greater than 0.05 were set as not significant (ns). Significance is notated with asterisks as follows: * p ≤ 0.05, ** p ≤ 0.01, and *** p ≤ 0.001. Statistical analysis and linear fitting was performed with OriginPro 2016. For statistics, while comparing two samples a Student’s t-test was employed, for comparison of three samples a one-way analysis of variance (ANOVA) with post-hoc Tukey pairwise comparison was used. Isoelectric points (IP) and molecular weight (MW) of proteins were calculated according to their specific protein sequence using the resources on www.expasy.org. Theoretical solubility of WNT agonist and purmorphamine was calculated in Perkin Elmer’s Chem3D 15.0 using the Molecular Networks plug-in for computation of LogS.

## Results and discussion

### Preparation and characterization of gelatin discs

Circular delivery discs with a diameter of 2 mm and a thickness of 2 mm were prepared as described (Fig 2A). Typically, the crosslinking of gelatin-based drug delivery systems such as microspheres are carried out in GA solution. However, in this study, GA vapor-phase crosslinking [31] was explored as this approach offered two clear advantages over solution-phase crosslinking: (1) gel swelling and deformation during crosslinking is avoided, and (2) removal of excess GA through washing steps is eliminated. Since swelling can affect the release of drug payload from the disc, the swelling behavior of discs crosslinked in GA vapor was compared to solution crosslinked discs by incubating them in PBS at 37°C for 2 months (Fig 2B). For comparison, solution-crosslinked discs were prepared in 0.25% GA (v/v) following a standard published protocol [23]. In general, the freeze-dried discs rehydrated in PBS within 24 h and maintained their physical dimensions throughout the 60-day period. Strikingly, vapor-phase crosslinked discs showed a 2-fold lower swelling by day 2 and furthermore, were stable up to 60 days, while solution phase crosslinked discs showed fluctuation in swelling between days 14–60. To ascertain the origins of the discrepancy in the swelling behavior, the quantification of free amino groups in gelatin, an indicator for the extent of crosslinking, was carried out using TNBS and normalized to untreated gelatin (Fig 2C). This showed that the crosslinking in the GA vapor-phase samples was higher compared to solution crosslinked discs. The higher swelling of solution crosslinked discs (800%) compared to vapor phase-crosslinked discs (500%) can therefore be attributed to the higher extent of crosslinking in the latter. Since uniformity in ultrastructure and macroscopic porosity is important for ensuring uniform drug diffusion, discs were lyophilized and their interior examined using SEM (Fig 2D). SEM images of the disc’s interior crosslinked either in vapor or solution revealed an interconnected porous structure with pore diameters ranging from 30 to 170 μm which should be conducive to protein diffusion [51]. The pore walls show a thickness between 5 and 10 μm. In crosslinked systems, the toxicity due to unreacted crosslinking agent is a concern. Unreacted glutaraldehyde can react with protein and as a leachant can be toxic to cells above certain concentration. While non-crosslinked discs showed no detectable level of aldehydes, discs crosslinked in GA vapor showed on average 350 ± 70 μM in the supernatant (S1 Fig). While this concentration could influence protein activity it is well below the cytotoxic threshold for [52] and furthermore, any unreacted glutaraldehyde would be expected to be neutralized through reaction with lysine residues in serum proteins. Additional confirmation for the cytocompatibility of the gelatin discs as delivery device was obtained by assessing the viability of hMSCs in presence of extracts of the gelatin disc using MTT assay (Fig 2E). In general, no toxicity towards hMSCs was observed up to 7 days. In sum, these observations suggest that GA vapor crosslinked gelatin discs are well suited for the delivery of molecules to cells.

### Delivery of small soluble signals

Loading of the freeze-dried gelatin discs was carried out using the well-established rehydration approach [53]. This approach offers one key advantage over loading before crosslinking of the gelatin disc, that is, the covalent linking of the soluble signal to the gelatin is eliminated. Two factors need to be considered while loading a drug into a gelatin matrix using the rehydration approach: (1) The isoelectric point of the gelatin and (2) the rehydration volume, which places a limit on the volume of liquid that can imbibed by the disc. Since gelatin type B has an isoelectric point of 5, the loading was carried out at pH 7.4 where the network is highly charged and should therefore be more conducive to water uptake. By placing the disc in a volume that is less than the hydration limit of the gelatin disc, complete absorption of the soluble signal was ensured.

**Fig 3:**
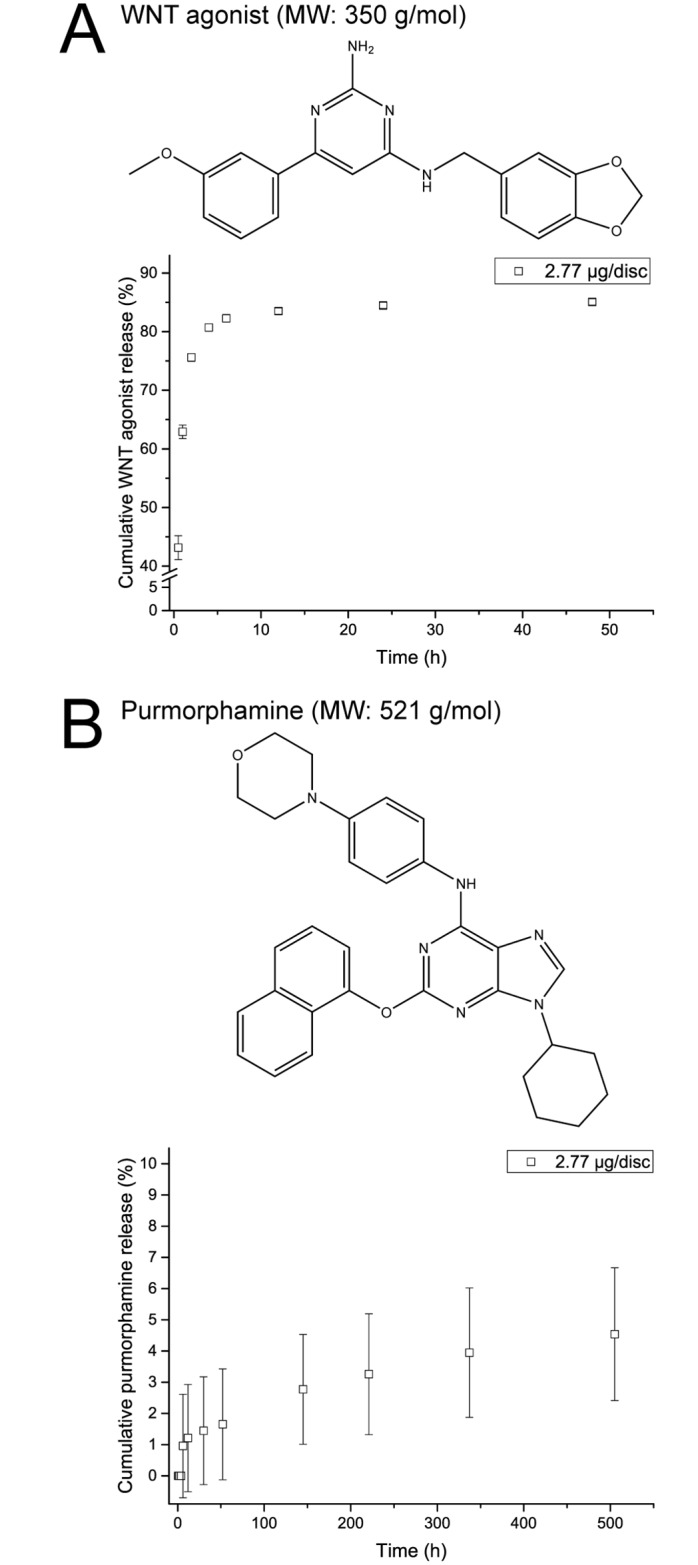
Quantification of WNT agonist and purmorphamine release. Cumulative release of WNT agonist **(A)** and purmorphamine **(B)** was determined by HPLC. Data is presented as mean ± SD, n = 3.

As a first step, the release of two small signaling molecules involved in EO, namely WNT agonist [36] and purmorphamine [38], an agonist of the hedgehog pathway, was quantified (Fig 3A and 3B) in order to demonstrate the release of a broad range of soluble signals. The choice of these two molecules was based on the fact that they differed in molecular weight, with purmorphamine being almost 170 Daltons larger than the WNT agonist (Table 1), and they act on different signaling pathways. Discs were loaded with 2.77 μg of WNT agonist or purmorphamine to ensure a release that is both biologically relevant as well as detectable using HPLC. While the WNT agonist showed a burst release during the first 2 h (76% of the payload) and reached a total cumulative release of 85.1 ± 0.5% after 48 h, purmorphamine showed no burst release and was released steadily over a period of 21 days with a total cumulative release of 4.5 ± 2.1%. This dramatic difference between the extent and duration of release between the WNT agonist and purmorphamine may be attributed to their different solubility in water. Due to the lack of solubility data for these signals, we calculated theoretical values. While the computed solubility of WNT agonist in water shows a LogS value of -5.04 which equals roughly 3.2 mg/l, the solubility of purmorphamine with a LogS of -9.03 (4.9×10^−4^ mg/l) is four orders of magnitude lower. The release of purmorphamine is therefore potentially limited by the solubility in aqueous release medium and not by its diffusion in the gelatin matrix. Nevertheless, these results demonstrated that the vapor-phase crosslinked gelatin discs were suitable as a matrix for release applications.

### Release of rhWNT3A and verification of bioactivity

Next we evaluated the loading and release of rhWNT3A, a protein implicated in controlling the fate of MSCs through regulation of self-renewal and apoptosis [54,55]. Despite its importance, literature on the release of rhWNT3A is limited to examples where the protein is physically bound to liposomes [56,57]. To the best of our knowledge, controlled delivery of free rhWNT3A from a delivery device has not been reported to date.

**Fig 4:**
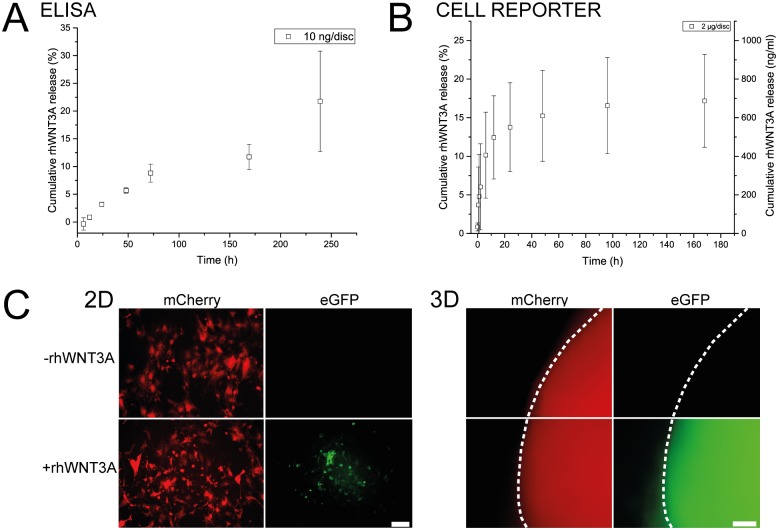
Quantification of rhWNT3A release. **A** Cumulative release of rhWNT3A as determined by ELISA. Values represent mean ± SD, n = 5. **B** Cumulative release of rhWNT3A as determined using the WNT cell reporter assay with mean ± SD, n = 3. While the primary axis describes the cumulative release in % of disc-loading, rhWNT3A concentration in the release medium is shown on the secondary axis. **C** Transduced WNT reporter hMSCs cultured in well plates (2D) and in pellets (3D) were exposed to rhWNT3A-loaded delivery discs and Wnt pathway activation was imaged using a fluorescence microscope. Transduced cells constitutively express mCherry and eGFP is expressed once the Wnt pathway is activated (scale bar: 100 μm). The pellet border is outlined with a white dashed line.

Discs loaded with 10 ng rhWNT3A showed a cumulative release of up to 21.8 ± 9.0% over a period of ten days as observed with ELISA (Fig 4A). The delivery profile showed initially a near linear release for the first three days followed by decreasing release for the remaining duration of the experiment. An important aspect that needs to be established during protein delivery is the retention of biological activity. While ELISAs are valuable tools for the quantification of proteins, they cannot be used to determine protein bioactivity. Depending on the epitope that is recognized by the antibodies, protein fragments could be detected and would lead to over- or under-estimation of the bioactive species. Therefore, we chose to confirm the bioactivity of rhWNT3A in a cell reporter assay.

An assay to determine both rhWNT3A bioactivity and concentration in solution, based on a WNT reporter cell line constitutively expressing mCherry and conditionally expressing eGFP on a WNT promoter, was thus developed in-house (see S1 Appendix and S2 Fig). The lower sensitivity of the cell reporter assay compared to the ELISA assay required higher rhWNT3A loading of the disc (200 x). The obtained release pattern was characterized by an initial burst release for the first 6 h which then transformed into a steadier release over time, resulting in a cumulative release of up to 17.2 ± 6.0% over a period of 7 days (Fig 4B). While the release patterns obtained through both assays seem comparable in terms of cumulative release and duration, a higher burst behavior was noted in the case of the cell reporter based assay, which might be due to the higher protein loading in this case. Yet, we do not necessarily expect congruence between the release pattern of rhWNT3A determined either by ELISA or the cell reporter system. Numerous factors influence intracellular Wnt signaling intensity as it is known to have negative feed-back regulation through the expression of AXIN2 [58,59]. It also appears that the fold change in β-catenin levels dictate Wnt signaling strength [60], which could also explain the early increase in intracellular Wnt signaling seen in the cellular reporter system. However, the overall similarity between ELISA and cell reporter results validates the gelatin matrix described herein for protein delivery.

To qualitatively test whether the rhWNT3A-loaded discs are able to invoke signaling in cells relevant for studying EO, a MSC-based WNT cell reporter system was used (Fig 4C). WNT reporter MSCs were cultured either in well plates (2D) or pellets (3D) and discs loaded with rhWNT3A or PBS (control) were added directly to the culture medium for a period of 72 h. In both 2D and 3D, eGFP expression in the samples treated with rhWNT3A was observed, indicating the activation of the WNT pathway through the released signal.

These results in sum represent the first example of rhWNT3A delivered from gelatin-based delivery discs and confirm the suitability of vapor-phase crosslinked gelatin for protein delivery in general.

### Release of rhFGF2 and verification of bioactivity

Encouraged by our findings that both small charged molecules and rhWNT3A can be released in a sustained manner, we next attempted the loading and release of rhFGF2. A key aspect of bone formation through EO is the recruitment of blood vessels and FGF2 plays a vital role in angiogenesis. Additionally, FGF2 governs proliferation and retention of differential potential in MSCs [61,62].

However, rhFGF2 presents new challenges with respect to sustained delivery as it is less stable at elevated temperatures [49]. For example, rhFGF2 activity at 37°C in DMEM is reduced by 85% within 24 h (S3A Fig). This loss of stability has been attributed to rhFGF2 aggregation [63].

A method that has been described for the stabilization of FGF2 is the formulation with heparin which leads to complex formation [49]. The addition of BSA to this formulation has been reported to have a positive effect on the release due to the inhibition of FGF2 adsorption to delivery devices made out of poly(lactide-co-glycolide) [48,64]. We therefore stabilized rhFGF2 with heparin and BSA in a ratio of 1:100:1000 with respect to rhFGF2. When this formulation was exposed to 37°C, the loss of detectable rhFGF2 was reduced to only 10% within the first 24 h (S3A Fig).

Since this approach was proven suitable for encapsulation in microspheres, we adapted this stabilization strategy to our gelatin discs. The challenge with implementing such a method using the gelatin disc system is the loading process through rehydration. All three macromolecular components have a different diffusion behavior due to their specific molecular weight and charge. Therefore, uniform loading of all three essential components cannot be guaranteed.

**Fig 5:**
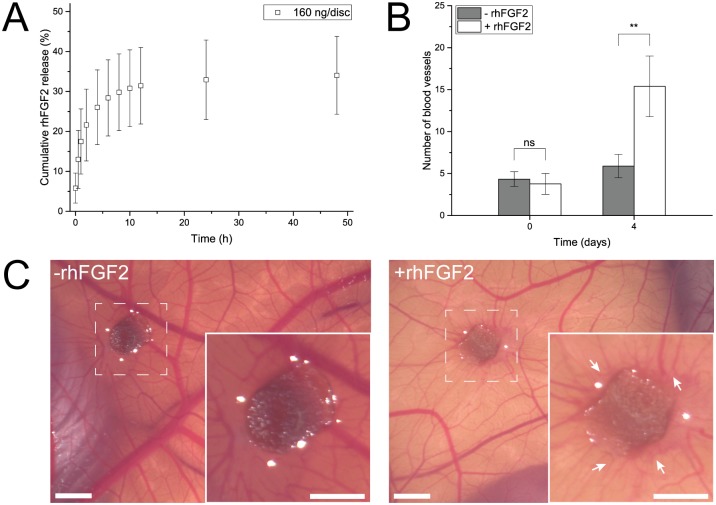
Release and bioactivity of rhFGF2. **A** Cumulative release from delivery discs. The amount of released rhFGF2 determined by ELISA. Data is presented as mean ± SD, n = 5. **B** Addition of rhFGF2-loaded discs (white) to CAMs led to increased blood vessel formation compared to control experiments (gray). Data is presented as mean ± SD, n = 4. **C** Exemplar pictures of the CAM assay. Left: Control discs without rhFGF2 induce no specific blood vessel formation. Right: rhFGF2-loaded discs lead to blood vessel formation (white arrows) towards the delivery disc. Insets: magnification of 2.2 x. All scale bars equal 2 mm.

In order to test the whole procedure, the formulated rhFGF2 was loaded to discs with a concentration of 160 ng/disc, which would permit relevant concentrations for cell culture and ELISA detection, and the release was carried out in DMEM cell culture medium. The release of rhFGF2 showed a biphasic pattern with an initial rapid release within 4 h followed by a lower release rate resulting in a final cumulative release of 34.0 ± 9.7% after 48 h (Fig 5A). Beyond 48 h, a negligible release in the range of 0.1% over 8 days (data not shown) was observed. Strikingly, release of unformulated rhFGF2 remained at levels that could not be quantified. These results thus highlight the importance of the stabilization as well as they show that this approach, despite of the potential loading problem, successfully leads to stabilization of rhFGF2 within the disc system.

To further confirm that the released rhFGF2 is biologically active, we used the well-established CAM assay wherein the placement of an angiogenic agent, in our case a rhFGF2-loaded gelatin disc, on the membrane of the chick embryo leads to induction of blood vessels [65]. We found that the rhFGF2 that was measured using ELISA was indeed biologically active. After four days, CAMs exhibited a statistically significant (P = 0.003) increased blood vessel formation of 260% when treated with rhFGF2-loaded discs. The growth of blood vessels was directed towards the delivery disc. This is in accordance with previously described results [65] and in contrast to experiments where CAMs were treated with free FGF2, resulting in global increase in vessel sprouting without any directionality [66]. In absence of rhFGF2, heparin and BSA-loaded discs (control) did not lead to increased blood vessel induction (Fig 5B). Exemplary microscopic pictures of both conditions are shown in Fig 5C.

### Release of rhVEGF and verification of bioactivity

**Fig 6:**
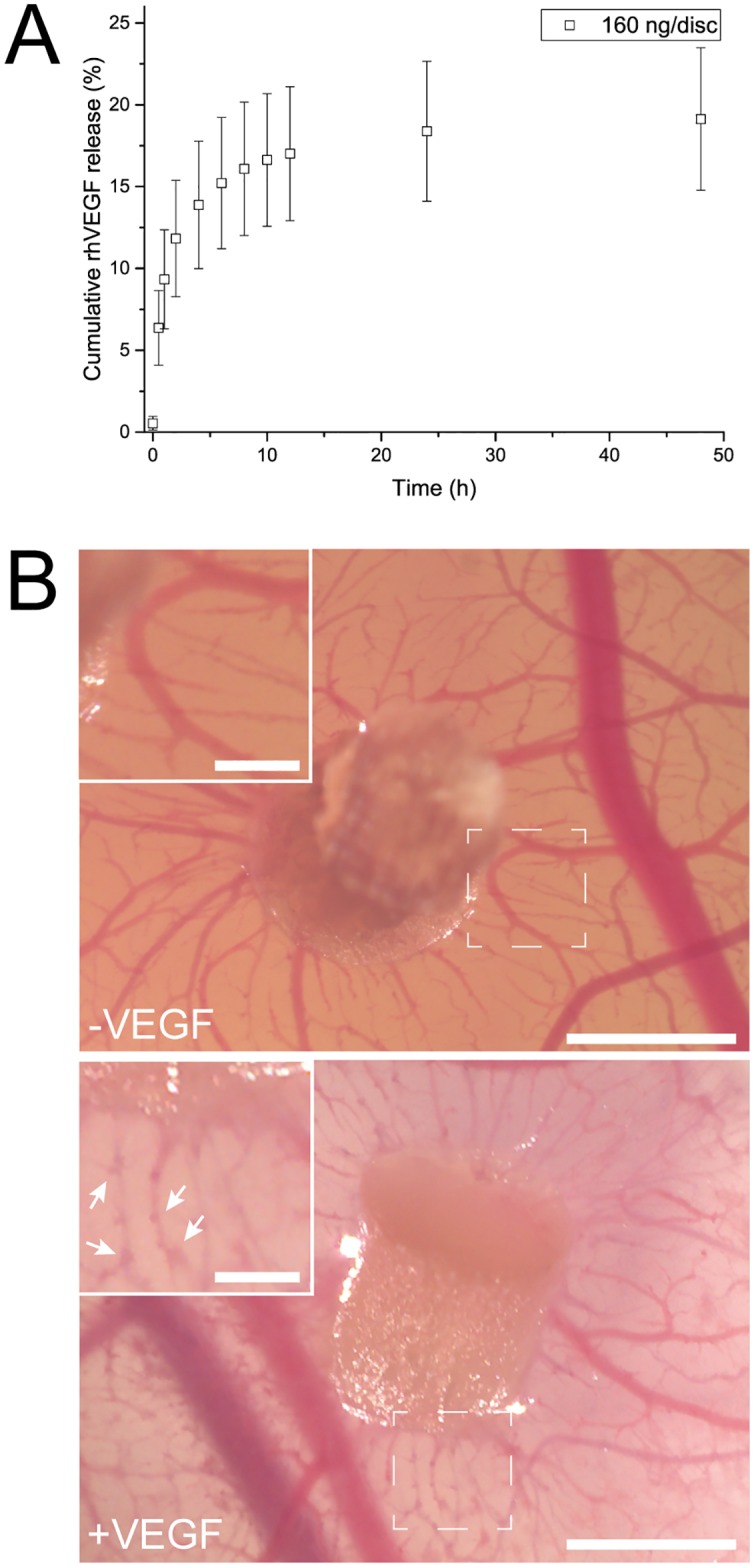
Release and bioactivity of rhVEGF. **A** Cumulative release of rhVEGF determined by ELISA. Data is presented as mean ± SD, n = 5. **B** In the CAM assay discs loaded with rhVEGF induced blood vessel sprouting (see white arrows in inset) (bottom) compared to non-loaded discs (top). Scale bar equals 2 mm. Scale bar in insets (2 x) equals 0.5 mm.

In addition to FGF2, VEGF plays a very important role in EO by promoting vessel sprouting and therefore branching [67] as well as in stabilization of blood vessels [68]. Therefore, the release of rhVEGF from gelatin discs was also explored. Gelatin discs were loaded with 160 ng of rhVEGF and the release of the protein was quantified over a period of 48 h using ELISA (Fig 6A). The obtained release profile resembled the release pattern observed for rhFGF2 with an initial burst release over the first 4 h followed by a steady state that lasted until 48 h beyond which it was undetectable using ELISA. Although, compared to rhFGF2 the cumulative release of rhVEGF is clearly reduced (19.1 ± 4.3%), this concentration range still is relevant for cell culture (Table 1).

To establish that the released rhVEGF is biologically active, we again employed the CAM assay as it is a well-established qualitative method to assess VEGF activity [69]. In this case, we looked at branching instead of *de novo* blood vessel formation. Indeed, we found that in absence of rhVEGF, blood vessels were formed in a uniform manner with a smooth vessel surface. In presence of rhVEGF however, the blood vessels were covered with small branching and protrusions and this indirectly confirmed, that the rhVEGF release from gelatin discs retained its bioactivity (Fig 6B). Thus the rhFGF2 and rhVEGF release taken in sum bode well for the use of gelatin discs for controlling angiogenesis, which are both pivotal events during EO.

### Release of rhBMP4 and verification of bioactivity

**Fig 7:**
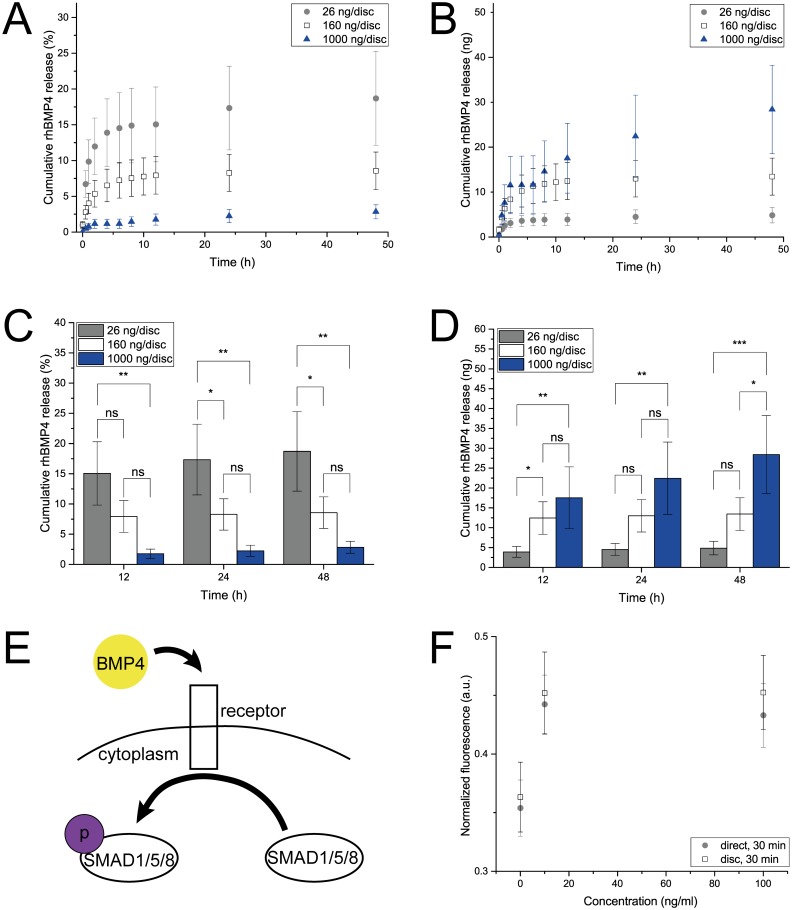
Release and bioactivity of rhBMP4. **A** and **B** Cumulative release of rhBMP4 from gelatin discs loaded with 26 ng (gray), 157 ng (white) or 1000 ng (blue) determined by ELISA and presented as % of disc loading and ng. Data is presented as mean ± SD, n = 5. **C** and **D** Statistical analysis of the rhBMP4 release from gelatin discs loaded with 26 (gray), 157 (white) or 1000 ng (blue) in % and ng with P≤0.05 equaling *, P≤0.01 equaling ** and P≤0.001 equaling *** and ns (not significant). **E** Schematic of the cellular reaction upon treatment with BMP4. **F** Medium conditioned with rhBMP4-loaded discs (white) induced phosphorylation of SMAD1/SMAD5 in hMSCs in a similar manner as direct addition of rhBMP4 to the culture medium did (gray). Phospho-SMAD1/SMAD5 concentration was determined by ELSIA. Data is presented as mean ± SD, n = °4.

After having successfully demonstrated the release of three proteins relevant for EO, we decided to explore the release of rhBMP4 from the gelatin delivery discs. BMP4 is part of the TGF-β signaling family and has an important role in promoting cartilage formation from mesenchymal cell condensates [70]. Especially interesting are the synergistic effects in combination with the three other proteins that have been shown to be compatible with our CRS: For instance, in combination with WNT3A, BMP4 induces early osteoblastic differentiation [71]; in combination with FGF2, it enhances osteogenic and chondrogenic differentiation of embryonic stem cells [72]; and, synergistically with VEGF, increases bone formation and bone healing [73]. Gelatin discs loaded with rhBMP4 (160 ng/disc) exhibited a maximal cumulative release of 8.6 ± 2.6% after 48 h (Fig 7A) which involves a burst release up to 4 h followed by a marginal release up to 48 h. Since rhBMP4 was released to a lesser extent than the other three proteins, we assessed whether rhBMP4 revealed similar thermal stability issues as rhFGF2. This was not the case as rhBMP4 as well as formulations containing heparin and BSA did not show any temperature-induced loss in activity over a period of six days at 37°C (S3B Fig).

Therefore, with the aim of increasing the release efficiency, discs loaded with different loadings (26 and 1000 ng/disc) of rhBMP4 were examined (Fig 7A and 7B). As expected, the cumulative release increased with a higher initial amount of rhBMP4 incorporation into the disc. Discs loaded with 26 ng delivered up to 5.0 ng in 72 h while discs loaded with 1000 ng released 32.6 ng in the same period (Fig 7B). However, the release efficiency did not linearly scale with the initial loading but rather showed a declining trend (Fig 7A). Discs loaded with 26 ng released up to 19%, while discs bearing 160 ng released up to 9%, and discs with a loading of 1000 ng rhBMP4 only released 3% of their initial content. After 48 h, a significant difference between most of the loading groups can be detected (Fig 7C and 7D). One plausible explanation is that at higher loading the entanglement between gelatin and protein chains increases and traps the soluble signal in the polymeric network, leading to a less efficient release process. Additionally, higher rhBMP4 concentrations might lead to protein aggregation within the delivery disc, an effect described for recombinant human albumin [74,75].

The binding of BMP4 to its cognate cell surface receptor induces an intracellular signaling cascade that involves the phosphorylation of SMAD1 and SMAD5 which are transcriptional factors downstream in the BMP pathway [76] (Fig 7E). Therefore, the bioactivity of rhBMP4 was examined using a phosphor-SMAD1/SMAD5 cell-based ELISA. Human MSCs, which express receptors for BMP4 [77], were exposed to different rhBMP4 concentrations either through the direct addition of rhBMP4 to the cell culture medium or by culturing the cells in release medium of rhBMP4-loaded discs. In both cases, phosphorylation of SMAD1 and SMAD5 increased by 25% compared to control experiments (Fig 7F). However, increasing the rhBMP4 concentration did not lead to higher phosphorylation. These results indicate that rhBMP4 delivered from gelatin discs remains bioactive since the degree of phosphorylation is similar to the samples where rhBMP4 was added directly.

The release characteristics of the four proteins evaluated in this study are summarized in Table 2. In all cases, only a fraction of the entire payload was released. This can be rationalized based on a combination of effects including electrostatic interactions (exclusion and association) between gelatin type B which has an IP of 5 and is therefore negatively charged at pH 7.4 and the positively charged signals, and molecular weight and flexibility of the proteins. For example, Tabata and Ikada demonstrated that gelatin type A, which is positively charged at physiological conditions, does not form these complexes with FGF2 as a model protein and therefore almost completely releases the payload over a short period of time [23]. In some instances a burst release might be favored as it can reduce the lag time between initial release and first cellular response. However, excessive release can also cause harmful concentration peaks and necessitate a more frequent change of delivery discs. Consequently, choosing type B gelatin and hence gaining some control over the release at the expense of reduced signal recovery was in this case deemed acceptable.

**Table 2:** Summary of the release characteristics of the delivered proteins. Discs were loaded with 160 ng/disc except for rhWNT3A where discs were loaded with 10 ng/disc (ELISA) and 2 μg/disc (CELL REPORTER).

Soluble Signal	Burst release duration (h)	Cumulative burst release (%)	Cumulative release after 48 h (%)
rhWNT3A			
ELISA	-	-	5.7 ± 0.5
CELL REPORTER	6	10.2 ± 5.6	15.2 ± 5.9
rhFGF2	4	26.0 ± 9.4	34.0 ± 9.7
rhVEGF	4	13.9 ± 3.9	19.1 ± 4.3
rhBMP4	4	6.5 ± 2.3	8.6 ± 2.6

## Conclusion

**Fig 8:**
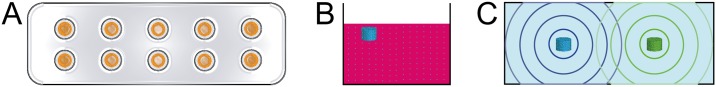
Possible applications of the gelatin delivery discs. **A** Prefabricated delivery discs could be stored in blister packs until further use, assuring protection against light and humidity. **B** Addition of discs to cell culture medium ensures controlled release of soluble signals over time. **C** Embedding one or multiple discs in a matrix material could lead to the formation of signal gradients in a 3D environment.

We demonstrate a novel and comprehensive *in vitro* delivery system for soluble signals involved in EO. By following our procedure, it is feasible to produce cheap, easy-to-use gelatin-based delivery discs in many desirable sizes that can be stored and used upon demand. Once a soluble signal is loaded, it can be released over a period of days while retaining its bioactivity. Possible applications are the direct addition into the culture medium of cells or the placement in another material such as Matrigel combined with the option to store ready-to-use discs packaged in hermetically sealed dispensers (Fig 8). The library of EO-associated soluble signals incorporated into our system thus far, *i*.*e*., rhFGF2, rhBMP4, rhVEGF, rhWNT3A, WNT agonist and purmorphamine, span a wide range of molecular weights and physicochemical characteristics. In the future one can envisage this repertoire of molecules to be extended to include parathyroid hormone-related protein or antagonists like Noggin [78] and Gremlin [79], further increasing the relevance of the proposed system. Also, the described setup allows recapitulating synergistic signaling effects. This is especially important since it becomes more and more apparent that signaling pathways are interacting with each other which leads to a more complex cellular behavior [80,81]. Combinations of soluble signals can either be achieved through the simultaneous use of different discs or the incorporation of several signals into the same disc. Preliminary results as well as the work of others [82,83] suggest that at least dual release from the same gelatin-based device is possible. Also, preliminary studies show that our system is capable of establishing signal gradients in a hydrogel environment.

## Supporting information

S1 Fig Residual glutaraldehyde concentration in crosslinked gelatin discs. Gelatin discs crosslinked in GA vapor showed non-toxic levels of aldehyde. Data is presented as mean ± SD, n = 3.
(TIF)

S2 Fig Description of the WNT3A cell reporter assay. **A** Transduced HEK 293 cells were seeded into well plates and incubated for two days. Culture medium was then replaced either with blanks (B), samples or a standard of known concentration, both in duplicates. After another 24 h, fluorescence expressed by the cells was determined. **B** Standard curve of the WNT assay ranging from 0 to 2000 ng/ml with mean ± SD, n = 20.
(TIF)

S3 Fig Stability of rhFGF2 and rhBMP4 at 37°C. **A** Decomposition of rhFGF2 under cell culture conditions within days can be avoided by formulating the protein with heparin and BSA (1:100:1000). **B** Stability of rhBMP4 remains constant over days and cannot further be improved by addition of heparin and BSA.
(TIF)

S1 Appendix Description of the WNT3A cell reporter assay. (DOCX)
