# Supplementary figures and images for: Gelatin device for the delivery of growth factors involved in endochondral ossification
Source: PLoS One. 2017 Apr 5;12(4):e0175095. doi: 10.1371/journal.pone.0175095 (PMC5381949; doi:10.1371/journal.pone.0175095)

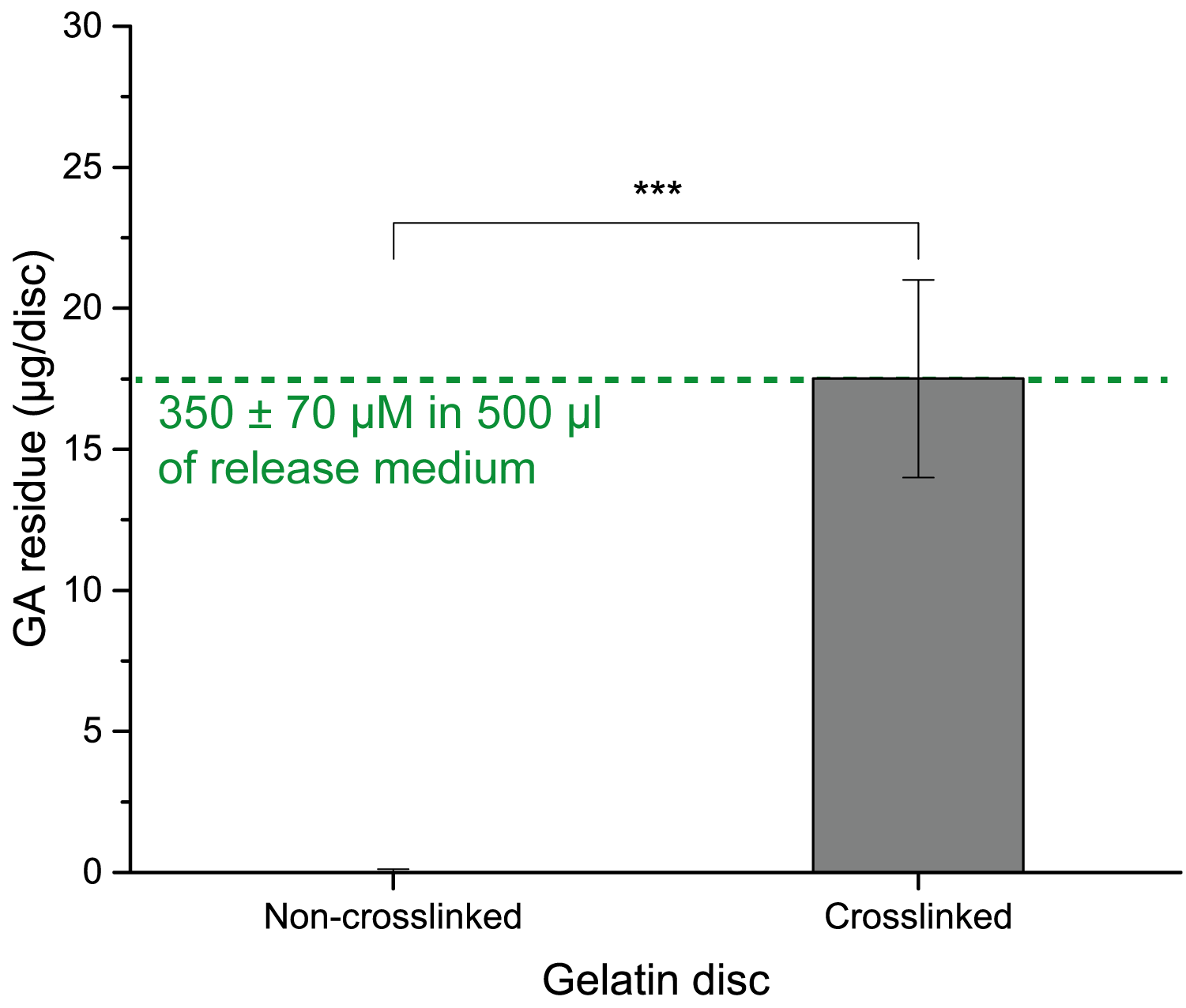

Supplement: S1 Fig — Gelatin discs crosslinked in GA vapor showed non-toxic levels of aldehyde. Data is presented as mean ± SD, n = 3. (TIF) [file pone.0175095.s001.tif]

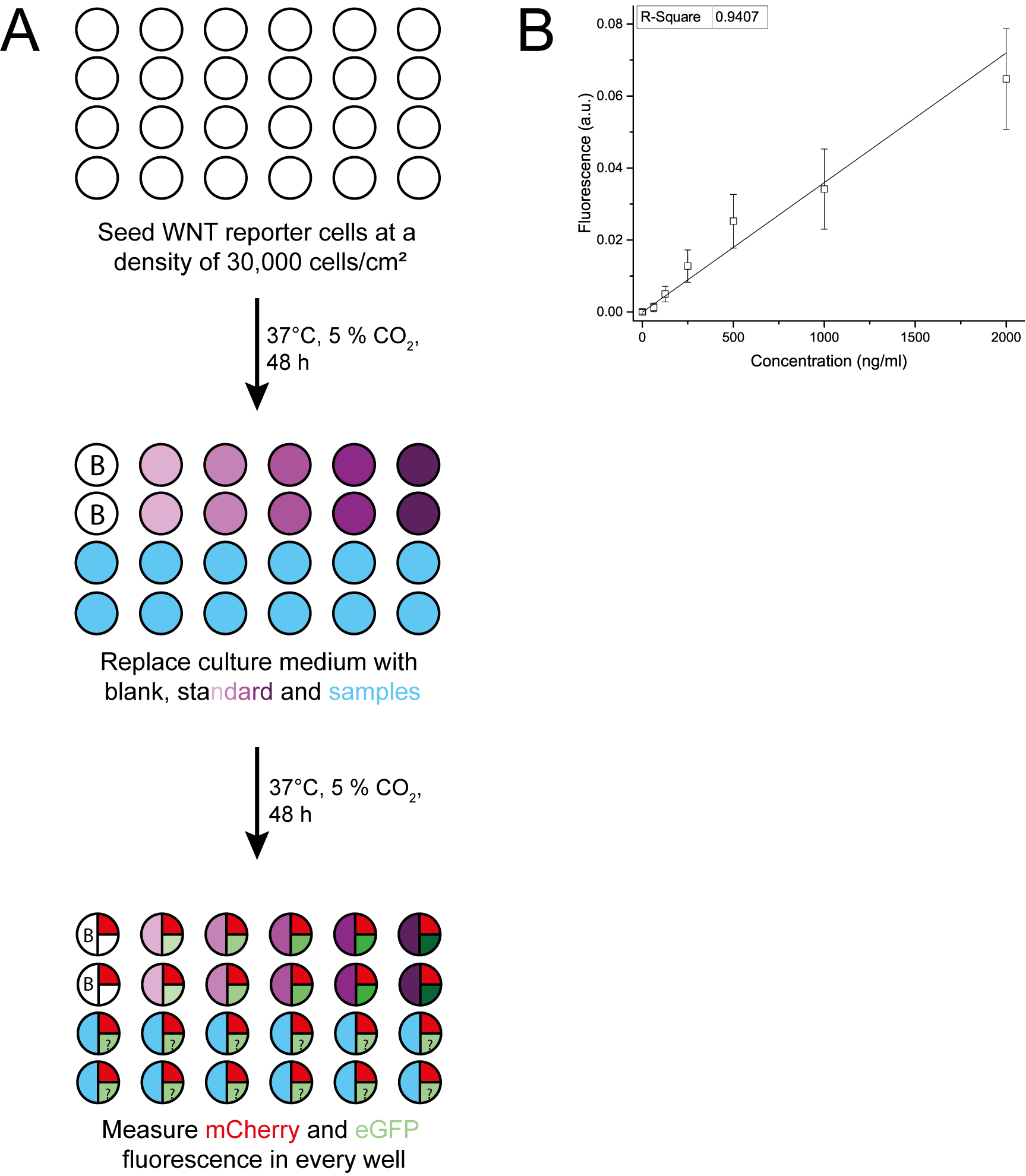

Supplement: S2 Fig — A Transduced HEK 293 cells were seeded into well plates and incubated for two days. Culture medium was then replaced either with blanks (B), samples or a standard of known concentration, both in duplicates. After another 24 h, fluorescence expressed by the cells was determined. B Standard curve of the WNT assay ranging from 0 to 2000 ng/ml with mean ± SD, n = 20. (TIF) [file pone.0175095.s002.tif]

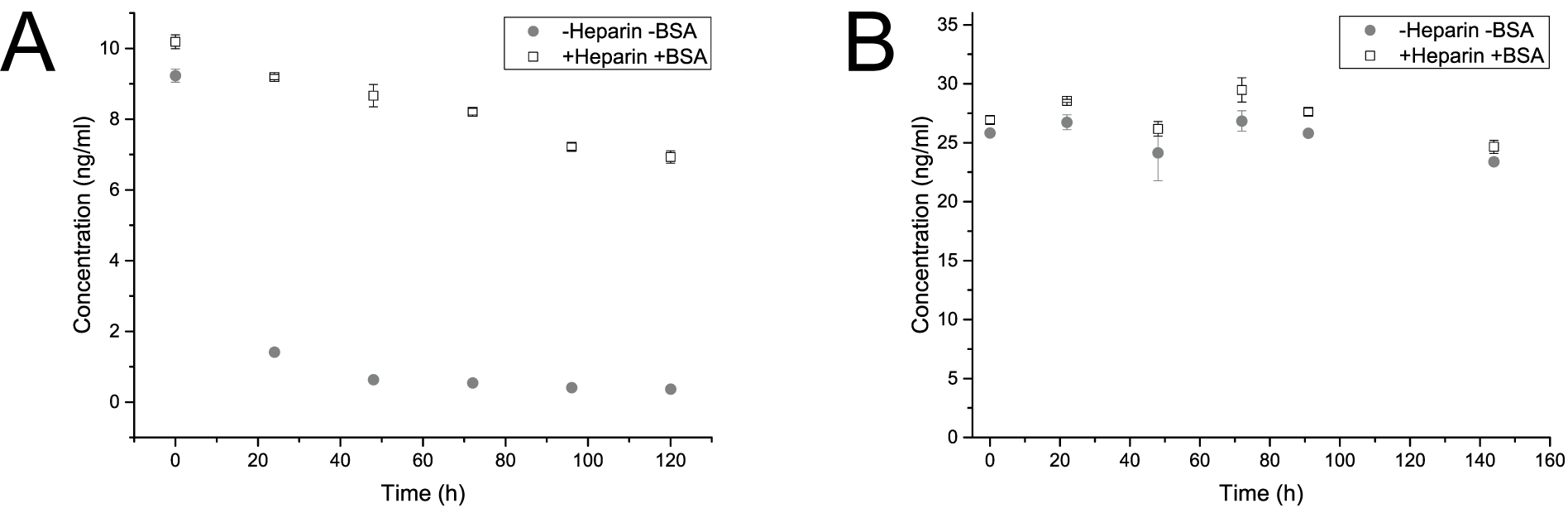

Supplement: S3 Fig — A Decomposition of rhFGF2 under cell culture conditions within days can be avoided by formulating the protein with heparin and BSA (1:100:1000). B Stability of rhBMP4 remains constant over days and cannot further be improved by addition of heparin and BSA. (TIF) [file pone.0175095.s003.tif]
