# Supplementary material for: Gelatin device for the delivery of growth factors involved in endochondral ossification
Source: PLoS One. 2017 Apr 5;12(4):e0175095. doi: 10.1371/journal.pone.0175095 (PMC5381949; doi:10.1371/journal.pone.0175095)
Supplement: S1 Appendix — (DOCX) [file pone.0175095.s004.docx]

**Supporting Information**

**WNT3A Cell Reporter Assay**

An assay to determine both rhWNT3A bioactivity and concentration in solution, based on a WNT reporter cell line constitutively expressing mCherry and conditionally expressing eGFP on a WNT promoter, was developed in-house. In this cell reporter assay, binding of rhWNT3A to its cognate receptor on HEK cells results in activation of the canonical Wnt pathway, which induces eGFP expression in the cells. The whole assay procedure is presented in S2A Fig. The fluorescence was quantified and normalized to untreated cell controls using fluorescence microscopy. The assay showed a linear dependency for rhWNT3A concentrations between 62.5 and 2000 ng/ml (S2B Fig). Concentrations below this threshold were neglected due to the limit of detection (blank value plus three times the blank’s standard deviation), while higher concentrations were not tested due to the lack of biological relevance.
